# Editorial: Understanding bladder tumor microenvironment to optimize immunotherapy

**DOI:** 10.3389/fimmu.2025.1566407

**Published:** 2025-02-17

**Authors:** Mathieu Rouanne, Laurent Derré

**Affiliations:** ^1^ Department of Microbiology & Immunology, Columbia University, New York, NY, United States; ^2^ Herbert Irving Comprehensive Cancer Center, Columbia University, New York, NY, United States; ^3^ Urology Research Unit and Urology Biobank, Department of Urology, Centre Hospitalier Universitaire Vaudois, Lausanne, Switzerland

**Keywords:** tumor microenvironment, immunotherapy, bladder cancer, mucosal immunity, anti-cancer therapy

## Introduction

Bladder cancer remains a public health concern due to its prevalence, high risk of recurrence and associated management costs. While significant advances have been made in the treatment of bladder cancer over the last five years, such as the new standard of care in the first-line treatment of metastatic bladder cancer with antibody-drug conjugate and PD-1 blockade, maintenance PD-L1 blockade and FDA approval of PD-1 blockade in the adjuvant setting after radical cystectomy for high-risk tumors, robust predictive biomarkers are still lacking. Currently, a multitude of novel immunostimulatory drugs, such as bispecific T cell engagers, CAR-T cells, recombinant interleukins, and oncolytic viruses are being tested in patients with bladder tumors. The clinical development of some novel immunomodulatory agents at earlier tumor stages of organ-confined disease is evolving rapidly. Understanding the roles, functions and responses of immune and non-immune cells in the bladder tumor microenvironment is critical to foster the successful development of immuno-modulatory drugs in non-metastatic bladder tumors.

In this cutting-edge Research Topic, our goal is to provide a discussion of the roles and mechanisms of immune and non-immune components of the bladder tumor microenvironment, from protective host immunosurveillance to the response to immunotherapy. We wish to encourage discussion and cross-fertilization between scientists, and clinicians among the various roles and aspects of the bladder tumor microenvironment and its responses to immune modulation, to further our knowledge in this field.

## The immune micro-environment of bladder cancer in the context of response to immune checkpoint inhibition

In this review, van Dorp et al. nicely described the growing field of treatments for muscle-invasive bladder cancer (MIBC). In particular, they discussed the promising results of immune checkpoint inhibitors (ICIs) targeting PD-1/PD-L1 and CTLA-4, which have shown breakthrough potential for bladder cancer patients over the past decade. The authors also highlighted recent clinical trials using ICIs in bladder cancer patients. Finally, the review emphasized the crucial role of immune checkpoints in anti-tumor response inhibition and how the interaction between the different cellular components of the tumor microenvironment may influence the effectiveness of ICIs. In this context, they also discussed the formation and role of tertiary lymphoid structures.

This review will guide the reader to important future research by providing a deeper understanding of the biological mechanisms underlying the use of ICIs in MIBC patients.

## Understanding bladder cancer risk: Mendelian randomization analysis of the influence of immune cells and inflammatory factors

Un et al. analyzed the association of immune cell populations, inflammatory cytokines and chemokines with bladder cancer. To do this, the authors used a research method called Mendelian randomization (MR). This method incorporates genetic variation as an instrumental variable to investigate the causal effects of immune cells and inflammatory cytokines on genetic susceptibility to bladder cancer, aiming to elucidate which immune cell subsets may be involved in the pathogenesis of bladder cancer. The authors examined publicly available data from genome-wide association studies (GWAS) that included single nucleotide polymorphisms (SNPs) profiling 731 immune phenotypes and 91 circulating inflammatory proteins from individuals of European ancestry The dataset included 2,053 bladder cancer cases and 287,137 controls. The authors identified five immunophenotypes associated with protective effects against bladder cancer – mainly activated CD8+ T cells and B cells. Their results also indicate that monocytes classified by CD14 and CD16 markers significantly contributed to bladder cancer progression. In addition, the authors highlighted the role of IL-20, a member of the IL-10 family, and IL- 22RA1, a component of its receptor, in bladder cancer development. Although *in vitro* and *in vivo* mechanistic studies are critically needed to validate the functional value of these immune components, this study offers new perspectives on the pathogenesis of the disease and potential therapeutic targets.

## Single-cell RNA sequencing analysis identifies acute changes in the tumor microenvironment induced by interferonα gene therapy in a murine bladder cancer model

Steinmetz et al. demonstrated that Nadofaragene firadenovec (Ad-IFNa/Syn3) – a currently approved interferon alpha gene therapy for BCG-unresponsive bladder cancer – has the potential to remodel the immune tumor microenvironment (TME) of the bladder. In the MB49 orthotopic model of bladder cancer, the authors evaluated changes in the tumor microenvironment following intravesical delivery of adenoviral vectors expressing murine IFNα (muAd-IFNα). Concomitantly with an upregulation of the immunogenic cell death signaling pathways, they showed a decrease in the Th2 pathway genes. They also described upregulation of PD1/PD-L1 pathways on macrophage populations in muAd-IFNα treated tumors compared to controls, most likely due to the release of IFN molecules in the TME. Finally, the authors also investigated the tumor cell and endothelial cell compartments of the bladder TME. They observed an upregulation of cell death pathways in both the tumor and endothelial clusters. Overall, this single-cell analysis reveals the early remodeling of the bladder tumor microenvironment 72 hours after intravesical delivery in the bladder and highlights putative mechanisms of action of this drug at an early time point.

## Case report: PD-L1-negative advanced bladder cancer effectively treated with anlotinib and tislelizumab: a report of two cases

In this case report, Li et al. described two patients with advanced and metastatic urothelial carcinoma who were treated with anlotinib and tislelizumab therapy as second-line therapy. Both patients had progressed after first-line chemotherapy with gemcitabine and cisplatin. Anlotinib is a tyrosine kinase inhibitor with anti-angiogenic and fibroblast growth factor receptor (FGFR) inhibitory effects. Tislelizumab is a programmed death-1 (PD-1) immune checkpoint inhibitor. Although both patients’ tumors were programmed death ligand-1 (PD-L1)-negative and only one patient had an FGFR3 mutation, both patients showed partial remission (12 months of progression-free survival). In addition, no significant side-effects were observed. Due to the current paucity of data on the combination of anlotinib and tislelizumab in advanced urothelial carcinoma, this study paves the way for further exploration of this combinatorial treatment strategy as a second-line therapy for advanced urothelial carcinoma.

## Concluding remarks

We thank the authors and dedicated reviewers for their efforts and enthusiasm. We believe that this Research Topic of reviews and preclinical and clinical studies is situated at the intersection of immunology and immunotherapy. We hope that this series of articles will stimulate and facilitate the communication between immunologists and clinicians in this rapidly evolving field.

